# Validation of Lung [^18^F]FDG Uptake as a Quantitative PET Biomarker for Influenza-Associated Pulmonary Inflammation

**DOI:** 10.1007/s11307-025-02051-y

**Published:** 2025-11-05

**Authors:** Carla Bianca Luena Victorio, Shantanu Gupta, Arun Ganasarajah, Joanne Ong, Ann-Marie Chacko

**Affiliations:** 1 Chacko Laboratory for Translational and Molecular Imaging, Cancer and Stem Cell Biology Programme, Duke-NUS Medical School Singapore, 8 College Road, Singapore, 169857 Singapore; 2 Division of Cellular and Molecular Research, National Cancer Centre Singapore, Singapore, 169610 Singapore

**Keywords:** [^18^F]FDG-PET, Molecular imaging, Influenza, Viral infection imaging, Lung inflammation, Imaging biomarker

## Abstract

**Purpose:**

Influenza (flu) is a respiratory illness caused by lung infection with influenza viruses. This study establishes lung [^18^F]FDG uptake by PET/CT as an accurate measure of lung inflammation associated with influenza A virus (IAV) H1N1 infection.

**Procedures:**

Immunocompetent BALB/c mice were infected with a highly lethal dose of influenza A virus (PR8 strain) and intravenously injected with [^18^F]FDG. *Ex vivo* tissue biodistribution was assessed by gamma counting, while *in vivo* tissue biodistribution was analyzed by VOI analysis of PET/CT images. Disease severity was also investigated by VOI measurements of high-resolution lung CT images. Infection and inflammation were confirmed by immunohistochemical staining; while viral replication and expression of inflammatory proteins (cytokines and chemokines) were measured in lung tissues by qRT-PCR and multiplex ELISA, respectively.

**Results:**

*Ex vivo* tissue biodistribution of [^18^F]FDG revealed that the lungs were the only relevant imaging target in influenza-infected mice. Lung [^18^F]FDG uptake on PET/CT images increased with disease severity and exhibited 1.53-fold increase on day 1 and up to 2.63-fold increase on day 6 post-infection compared to pre-infection levels. Lung uptake correlated with the increased production of pro-inflammatory proteins associated with influenza infection.

**Conclusions:**

Lung [^18^F]FDG uptake on PET images is a non-invasive molecular biomarker of influenza-A virus-induced lung inflammation and disease, effectively distinguishing infected from non-infected lungs as early as day 1 post-infection.

**Supplementary Information:**

The online version contains supplementary material available at 10.1007/s11307-025-02051-y.

## Introduction

Seasonal influenza (flu) is a respiratory illness caused by human influenza viruses circulating annually in the population, which typically results in mild, self-limiting disease. However, seasonal flu can also lead to an estimated 3–5 million severe lower respiratory illnesses requiring hospitalization, and approximately 400,000 deaths worldwide each year across all age groups [1–3]. In addition, seasonal flu accounts for 16.7 million disability-adjusted life-years (DALYs) globally, with 10.9 million DALYs specifically in children under 5 years of age [4]. DALYs are calculated by combining years lived with disability (YLD) and years of life lost (YLL) due to premature death. Out of 85 pathogens evaluated in 2019, influenza ranks as the 11th leading cause of DALYs across all age groups and the 7th leading cause of DALYs among children under 5 years [4].

The burden of seasonal flu extends beyond respiratory illness. For instance, patients with diabetes mellitus experience more severe influenza-related lower respiratory complications and higher mortality rates compared to non-diabetic individuals (reviewed in [5, 6]). Acute influenza infections can also lead to neurological complications including seizures, encephalopathy, extremity paresis, and Guillain-Barré Syndrome due to neuroinflammation [7]. Additionally, influenza infections are associated with an increased risk of ischemic heart disease globally [8]. These factors highlight the significant public health burden of seasonal flu and underscore the need for effective therapeutics to mitigate severe disease outcomes.

One way to expedite the clinical translation of influenza therapies is by using non-invasive biomarkers to monitor disease progression and predict recovery following therapeutic intervention. Traditional blood biomarkers [9, 10] and volatile organic compounds excreted via respiration (breath biomarkers) [11, 12] can indicate the severity of influenza infection but do not enable spatial localization of the infection. In contrast, pathogen-specific imaging techniques—such as direct virus labeling [13] or genetic engineering of virus to express bioluminescent proteins [14–17]—can localize infections in tissues but do not provide *in situ* molecular insights into disease mechanisms. Further, optical imaging modalities like bioluminescence are limited by the poor tissue penetration of light. Therefore, it is crucial to identify and characterize imaging biomarkers that can both track the spatiotemporal dynamics and reveal the molecular pathogenesis of infections [18]. These challenges are addressed by non-invasive nuclear imaging, particularly positron emission tomography (PET) using [^18^F]fluorodeoxyglucose ([^18^F]FDG).

[^18^F]FDG-PET imaging has been widely used to study viral infections in animal models, including monkeypox [19], severe fever with thrombocytopenia syndrome (SFTSV) [20], and respiratory viruses such as Middle East Respiratory Coronavirus (MERS-CoV) [21], and SARS-CoV-2 [22]. Our research group has also demonstrated [^18^F]FDG uptake in the intestines of dengue virus-infected mice [23] and in the lymphoid tissues of Zika-infected mice [24], with uptake correlating to inflammation and disease severity. Previous incidental findings of elevated lymphoid [^18^F]FDG uptake following influenza vaccination [25–27], as well as in cancer patients undergoing routine PET scans [28], suggest a potential role for [^18^F]FDG-PET in identifying influenza infections. Additionally, increased [^18^F]FDG lung uptake has been reported to correlate with disease severity in a ferret model of 2009 pandemic H1N1 influenza [29, 30]. However, a systematic evaluation of [^18^F]FDG lung uptake in relation to the extent of pulmonary inflammation status has not been performed. In this study, we provide direct evidence validating lung [^18^F]FDG uptake as a robust PET imaging biomarker for detecting *in situ* lung pulmonary inflammation associated with influenza infection in a mouse model of influenza A virus (IAV) H1N1 infection.

## Materials and Methods

### Materials

Female BALB/c mice, aged 3–4 weeks, were procured from In Vivos Pte. Ltd. (Singapore). The influenza A virus (IAV) H1N1 strain (A/Puerto Rico/8/34), commonly known as PR8, was obtained from the American Type Culture Collection (ATCC, USA). [^18^F]FDG with > 95% radiochemical purity was supplied by Singapore Radiopharmaceuticals Pte. Ltd. (Singapore).

### Influenza A Virus (IAV) Murine Infection Model

The animals were housed in individually ventilated cages and given standard mouse chow and water *ad libitum*. Infections were performed as previously described [31]. On the day of infection, mice were anesthetized by intraperitoneal administration of ketamine (70 mg/kg) and xylazine (15 mg/kg) cocktail and were placed in dorsal recumbency. The virus inoculum (10^7^ TCID_50_ PR8 in 30 μL) representing 1,000x 50% humane endpoint dose (HD_50_) was slowly instilled into the left nostril. Mice were monitored daily, and their weights recorded. Mice that lost > 20% of the original body weight were euthanized. All procedures adhered to applicable institutional and national guidelines for animal care and use.

### *Ex Vivo* Lung Pathology

Immunohistopathological examination of the lungs was performed as previously described [32]. Freshly harvested lungs were fixed in 10% neutral buffered formalin, processed for paraffin embedding, and sectioned into 5 µm slices. Contiguous tissue sections were stained with hematoxylin & eosin (H&E) and subjected to immunofluorescence (I.F.) staining for IAV nucleoprotein (Invitrogen cat. no. PA17221) and translocator protein (TSPO) (Invitrogen cat. no. MA5-31,966) (ThermoFisher Scientific, USA). I.F. tissue sections were counterstained with DAPI, and autofluorescence was quenched using the with Vector® TrueVIEW® kit (Vector Laboratories, USA).

Flash-frozen lung tissues (~ 30 mg) were homogenized in phosphate-buffered saline (PBS, pH 7.4) containing a protease/phosphatase inhibitor cocktail (Sigma-Aldrich, USA). After centrifugation at > 16,000 ⨉*g* to clear the homogenate, the supernatant was supplemented with Triton X-100 to a final concentration of 1% (^v^/_v_) and subjected to one freeze–thaw cycle. Samples were clarified, and total protein concentrations were determined using a BCA assay. Total protein extracts were analyzed by Luminex multiplex ELISA according to the manufacturer’s instructions (ThermoFisher Scientific, USA).

Total RNA was extracted from frozen lungs using RNeasy Mini Kit (Qiagen, Germany) according to the manufacturer’s instructions. Quantification of influenza A virus RNA using primers INFA-1 and INFA-2 as described previously [33] was performed by one-step reverse transcription quantitative PCR (RT-qPCR) Luna Universal One-Step RT-qPCR Kit (NEB, Cat. #E3005E). Absolute quantification of copy numbers was performed using influenza A/PR/8/34 genomic RNA standards (ATCC VR-1469DQ).

### Study Design

Three cohorts were employed in this investigation (Fig. 2). In the first study cohort, mice were subjected to *ex vivo* [^18^F]FDG tissue biodistribution (BioD) and digital autoradiography (DAR) studies on various days post-infection to dissect [^18^F]FDG tissue retention and identify relevant tissues for PET imaging. In the second cohort, mice were subjected to serial [^18^F]FDG-PET/CT imaging at pre-infection and on days 1, 3, and 5 post-infection to investigate temporal changes in lung [^18^F]FDG uptake resulting from influenza infection. Mock-infected mice were also imaged on the same days as comparators. Finally, in the third cohort, infected mice underwent cross-sectional [^18^F]FDG-PET/CT at specific days from baseline to day 6 post-infection, and tissues were collected immediately post-mortem for fully paired *ex vivo* assessments—including tissue viral load determination and inflammatory response assays. Cohort 3 was used for validation studies to confirm whether lung [^18^F]FDG uptake was a proxy of lung influenza infection and inflammation in this model.

### *Ex Vivo* [^18^F]FDG Tissue Retention

Tissue biodistribution (BioD) studies and autoradiography (DAR) imaging were performed as previously described [34]. Briefly, 20–30 MBq [^18^F]FDG (100 µL) was injected intravenously into mice at pre-infection, and on days 2, 4, and 6 post-infection. After 60 min uptake and distribution phase, blood was removed by cardiac puncture from deeply anesthetized mice. Subsequently, mice were euthanized, and relevant tissues were collected, weighed, and subjected to DAR imaging and gamma counting. For DAR, tissues were exposed to multi-purpose phosphoscreen (BAS-IP MS; GE Healthcare Life Sciences, USA) for 30 min. The screens were then scanned using the Sapphire Biomolecular Imager (Azure Biosystems, USA) at a resolution of 100 µm.

### [^18^F]FDG-PET/CT Image Acquisition and Reconstruction

[^18^F]FDG-PET/CT imaging was performed as previously described [24]. A dose of 20–30 MBq [^18^F]FDG (100 µL) was injected intravenously. After 60 min of uptake, mice were sedated with 2% isoflurane, and four mice were simultaneously imaged using PET/CT, focusing on the thorax, for 20 min using Vector^4^CT scanner (MILabs, Netherlands) with a HE-GP-RM 3.6 mm collimator. This was followed by a 5 min total-body CT scan (0.24 mA tube current, 50 kV tube voltage). PET images were reconstructed at 0.8 mm voxel resolution using a SROSEM algorithm with 16 subsets and 24 iterations, followed by a 1.5 mm Gaussian filter. Attenuation-corrected PET images and CT images were automatically co-registered in the MILabs image reconstruction software.

### Image Data Analysis

Autoradiography images were processed and analyzed using ImageJ (National Institutes of Health (NIH), USA). PET and CT image analyses were performed using PMOD version 4.4 (PMOD Technologies, Switzerland). Lung volumes of interest (VOIs) were created as previously described [35]. VOIs were manually delineated around the ribcage using CT images converted to HU values and refined using the cold iso-contouring function with a maximum threshold of −300 HU. These VOIs were then used to quantify lung [^18^F]FDG uptake on PET images and healthy lung volumes on CT images.

Metabolic lung volume (MLV) was calculated by determining the volume of lung VOI with [^18^F]FDG uptake exceeding the basal threshold. The basal threshold was defined by the formula: mean + 2x(standard deviation) of lung [^18^F]FDG uptake values from pre-infection (baseline) data. In PMOD, the MLV was calculated on PET images using the hot iso-contouring function with the calculated threshold as the minimum value.

### Statistical Methods

All statistical analyses and data visualizations were performed using Prism version 10.2 (GraphPad Software, USA). Comparison between two groups were compared with Welch’s *t*-test, while comparisons among more than two groups were performed with Welch’s ANOVA. Post-hoc statistical analyses were performed to establish effect size (*d*) and critical *t* (*t*_*c*_) values using G*Power ver.3.1.9.7 (Universität Kiel, Germany).

## Results

### Lung Inflammation Is a Hallmark of the Influenza A Infection Model

**Fig. 1:**
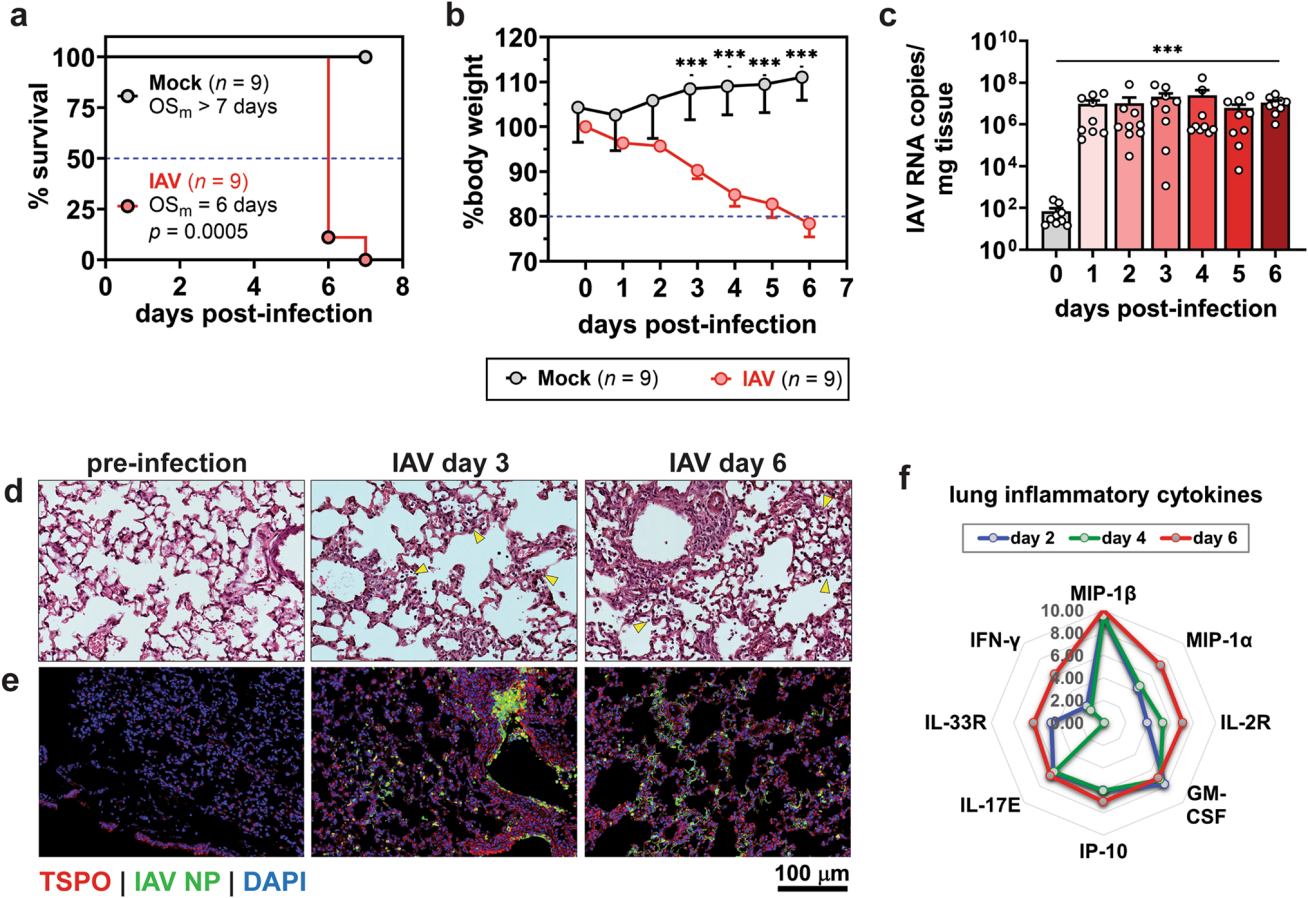
Lung inflammation in a lethal mouse model of influenza A (IAV) infection. **a** Kaplan–Meier survival curves compared by Mantel-Cox test. **b** Body weights compared by unpaired Welch’s t-test. **c** Tissue viral loads measured by absolute quantitative RT-PCR of lung tissues. Data are shown as mean ± SEM (*n* = 9 per day) from two independent studies, and means are compared by Welch’s ANOVA test. **d** Representative lung tissue sections stained with hematoxylin and eosin. (**e**) Representative immunofluorescence staining of lung sections for influenza A virus nucleoprotein (IAV NP) and translocator protein (TSPO) inflammation biomarker. **f** Radial plot depicting temporal pattern of pro-inflammatory cytokine and chemokine expression in homogenized influenza-infected lungs (*n* = 6 per day). Data from the eight most highly expressed proteins are shown as log_2_(fold-change mean expression relative to pre-infection) from two independent studies. * *p* < 0.05. ** *p* < 0.005. *** *p* < 0.001

Mice infected with influenza A virus (IAV) PR8 strain developed lethal disease, with a median overall survival of 6 days (Fig. 1a). Mice were euthanized from day 6 onwards due to significant weight loss (> 20%) (Fig. 1b) but with no other overt signs of illness. Necropsy revealed a dramatic increase in viral RNA levels in the lungs, with a 100,000-fold rise from day 1 compared to pre-infection (Fig. 1c), which indicated rapid viral replication. Histopathological examination further confirmed lung infection and inflammation. H&E staining of lung tissue sections exhibited immune cell infiltration into the lung parenchyma and alveolar spaces (Fig. 1d). Immunofluorescence staining for influenza nucleoprotein (NP) and translocator protein (TSPO)—an inflammation marker on immune cells—demonstrated immune cell aggregates in areas of high viral NP expression (Fig. 1e). Additionally, influenza infection resulted in elevated expression of inflammatory cytokines and chemokines. A radial plot of the temporal expression pattern of the eight most highly upregulated proteins revealed increased expression as early as day 2, peaking on day 6 (Fig. 1f). These findings provide strong evidence of a heightened inflammatory response in the lungs of PR8 influenza A-infected mice.

### The Lung Is the Primary Tissue of [^18^F]FDG Retention in Influenza-Infected Mice

**Fig. 2:**
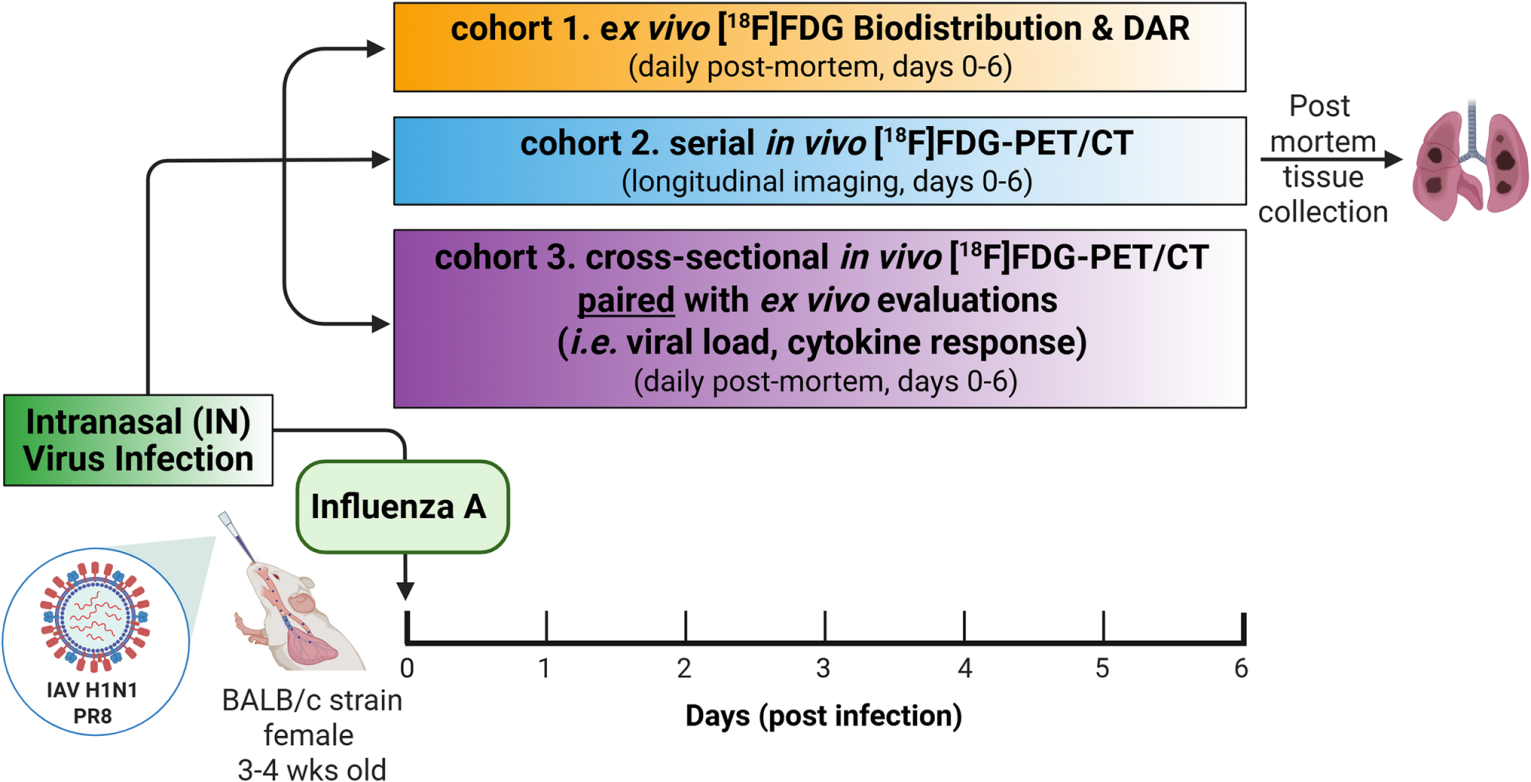
[^18^F]FDG Study timeline and experimental design. The study was conducted with three independent cohorts of Influenza A (IAV) infected mice. Cohort 1 was used for *ex vivo* tissue biodistribution (BioD) and digital autoradiography (DAR) studies to dissect [^18^F]FDG tissue retention. Infected animals were subjected to necropsy and tissue BioD assays on various days post-infection. Cohort 2 was subject to serial [^18^F]FDG-PET/CT imaging to evaluate temporal changes in lung [^18^F]FDG uptake during disease. Cohort 3 was used for cross-sectional [^18^F]FDG-PET/CT imaging on various days post-infection and paired with *ex vivo* evaluation of viral replication and inflammatory responses. Image created with BioRender

**Fig. 3:**
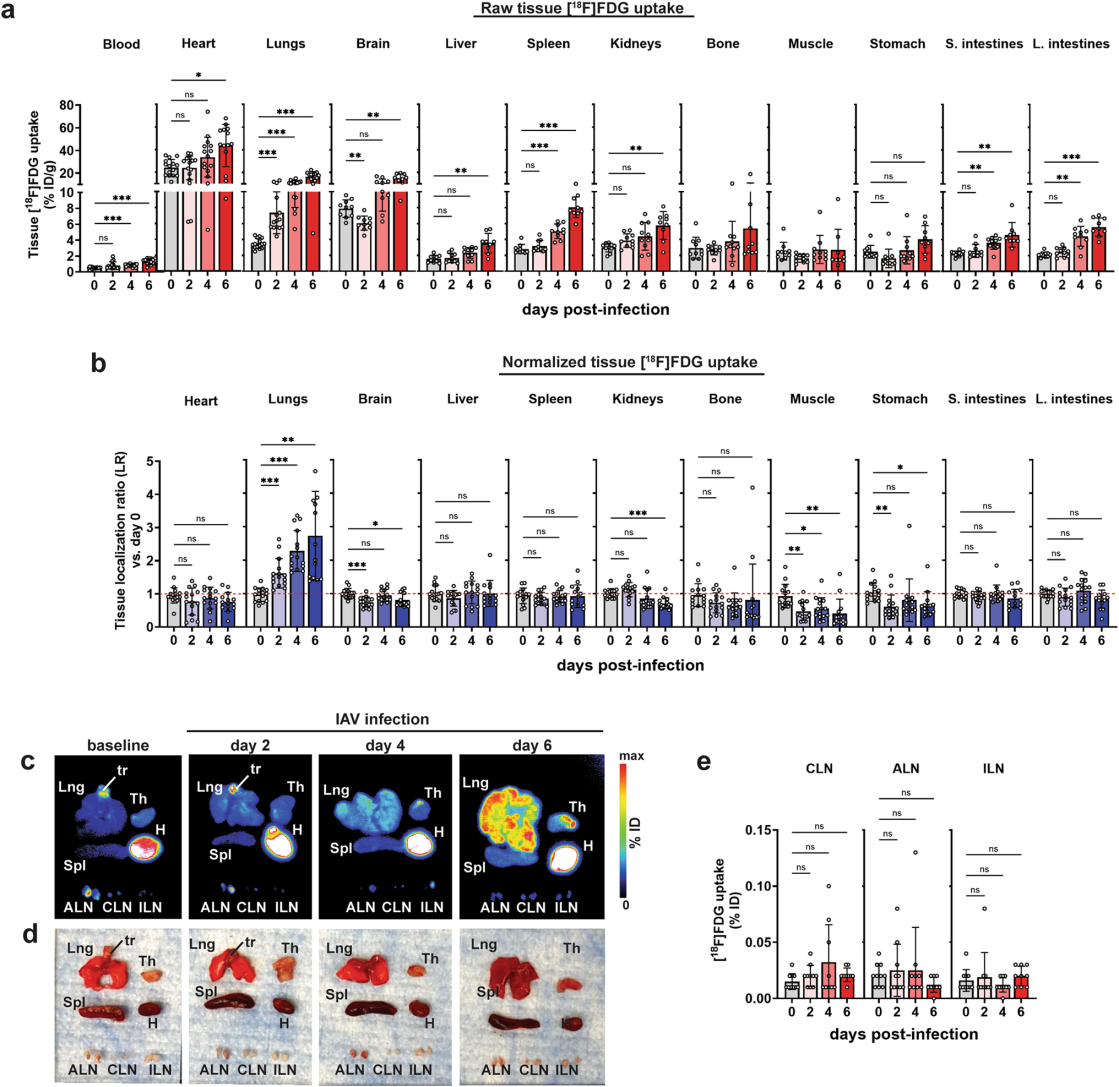
*Ex vivo* biodistribution of [^18^F]FDG in influenza infected mice. **a **Raw tissue [^18^F]FDG uptake reported as %ID/g and **b **Normalized tissue [^18^F]FDG uptake reported as tissue localization ratio (LR). LR was calculated by normalizing ${~}^{{\mathrm{lung}}}\!\left/ \!{~}_{{\mathrm{blood}}}\right.$ uptake ratio in disease with ${~}^{{\mathrm{lung}}}\!\left/ \!{~}_{{\mathrm{blood}}}\right.$ uptake ratio at pre-infection. **c** Autoradiographs of selected tissues at 60 min post-injection with [^18^F]FDG are shown alongside same tissues photographed prior to autoradiography (**d**). **e** [^18^F]FDG uptake in selected lymph nodes is reported as %ID. Data are shown as mean ± SEM (*n* = 10 mice/day) from three independent studies. Means are compared by Welch’s ANOVA. * *p* < 0.05. ** *p* < 0.005. *** *p* < 0.001. *ns*, not significant. *Lng*, lungs. *tr*, trachea. *Th*, thymus. *H*, heart. *Spl*, spleen. *ALN,* axillary lymph nodes. *CLN*, cervical lymph nodes. *ILN,* inguinal lymph nodes

To identify which tissues exhibit increased [^18^F]FDG uptake following influenza A infection, we performed *ex vivo* tissue biodistribution assays at various days post-infection (Fig. 2). As expected, [^18^F]FDG retention in the lungs on day 2 post-infection (5.92 ± 0.88%ID/g) was 1.7-fold higher (*p* < 0.001) compared to pre-infection levels (3.48 ± 0.65%ID/g) (Fig. 3a, Table 1). The retained [^18^F]FDG in the lungs continued to increase until day 6 (18.40 ± 2.25%ID/g), which was 5.3-fold higher (*p* < 0.001) than pre-infection (Fig. 3a, Table 1). In addition to the lungs, several other organs, including the heart, brain, liver, spleen, and intestines, also exhibited markedly elevated [^18^F]FDG compared to baseline (Fig. 3a). Notably, [^18^F]FDG activity in the blood was higher on day 4 (0.81 ± 0.18%ID/g) and day 6 (1.43 ± 0.31%ID/g) post-infection, compared to pre-infection levels (0.52 ± 0.08%ID/g) (Fig. 3a, Table 1). Normalization of tissue [^18^F]FDG retention with residual blood levels and baseline pre-infection levels at day 0 (*i.e.,* localization ratio; LR) revealed a time-dependent increase in [^18^F]FDG retention only in the lungs, indicating selective pulmonary involvement during influenza infection (Fig. 3b). Relative to pre-infection, LR was 1.51-fold (*p* < 0.001), 2.05-fold (*p* < 0.001), and 2.31-fold (*p* = 0.001) higher on days 2, 4, and 6 post-infection, respectively (Table 1). These results were further corroborated by autoradiographs depicting progressively elevated [^18^F]FDG retention in the lungs that occurred with increasing disease severity (Fig. 3c-d). Neither the spleen (Fig. 3b, c) nor the lymph nodes (Fig. 3b, c, e) manifested any significant changes in [^18^F]FDG retention patterns after influenza infection. Together, these results highlight the lungs as the primary and most relevant imaging target for *in vivo* [^18^F]FDG-PET imaging in this influenza infection model.

**Table 1:** [^18^F]FDG uptake in lungs and blood from influenza A virus (IAV)-infected mice determined by *ex vivo* gamma counting

Days post-infection	FDG Lung Uptake		FDG Blood Uptake		Lung Uptake/Blood Uptake	
	%ID/mL	*p*-value^b^	%ID/mL	*p*-value^b^	LR^c^	*p*-value^b^
**0**^a^	3.48 ± 0.65		0.52 ± 0.08		1.00 ± 0.30	
**2**	5.92 ± 0.98	< 0.001	0.60 ± 0.14	ns	1.51 ± 0.32	< 0.001
**4**	10.98 ± 1.98	< 0.001	0.81 ± 0.18	< 0.001	2.05 ± 0.29	< 0.001
**6**	18.40 ± 2.25	< 0.001	1.43 ± 0.31	< 0.001	2.31 ± 0.83	0.001

Data are expressed as mean ± S.D. (*n* = 10 mice/day)

^a^Day 0 refers to pre-infection

^b^Mean uptake values were compared to Day 0 with Welch’s ANOVA

^c^LR refers to lung/blood localization ratio

### Lung [^18^F]FDG Uptake Is an Early *In Vivo* Imaging Biomarker of Influenza Lung Inflammation

**Fig. 4:**
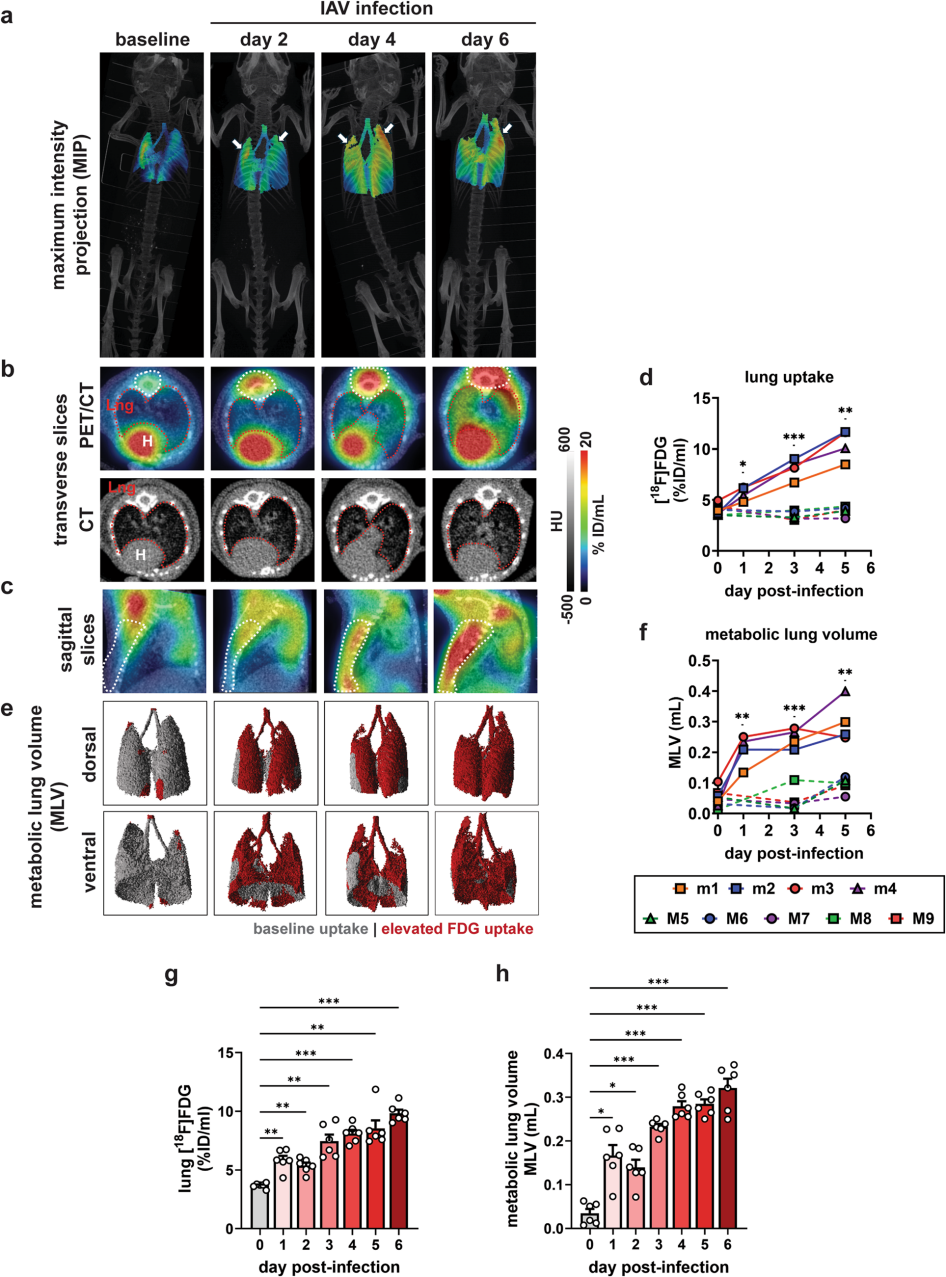
[^18^F]FDG-PET/CT imaging of influenza infected lungs. **a** Representative maximum intensity projection (MIP) images of lung-focused [^18^F]FDG-PET and whole-body CT from longitudinal scans (*n* = 5). [^18^F]FDG uptake outside the lungs are masked. **b** Transverse PET/CT and CT image slices. The lung VOIs are shown in red dashed lines. Spinal uptake reminiscent of reactive marrow hyperplasia are shown in dashed white lines. *Lng*, lungs. *H*, heart. **c** Sagittal PET/CT image slices highlighting reactive marrow hyperplasia in white dashed lines. **d** Quantification of lung FDG uptake by VOI analysis from a representative cohort of Mock-infected (M1-M5) and PR8-infected (m1-m4) mice serially imaged from pre-infection to day 5 post-IAV infection. **e** Surface-rendered 3D representation of metabolic lung volumes (MLV). Basal [^18^F]FDG uptake is shown in grey, while elevated [^18^F]FDG uptake representing inflammation is shown in red. **f** Quantification of MLV by VOI analysis. **g**-**h** Quantification of (**g**) lung [^18^F]FDG uptake and (h) MLV data from cross-sectional [^18^F]FDG-PET imaging (*n* = 6 per imaging day). Data are shown as mean ± SEM representative of three independent studies. Means were compared using Welch’s ANOVA. * *p* < 0.05. ** *p* < 0.005. *** *p* < 0.001

Serial PET/CT imaging of the thoracic region in influenza-infected mice (Fig. 2) revealed diffuse, globally increased [^18^F]FDG uptake in both lungs with some foci of increased signal longitudinally, as shown in maximum intensity projection (MIP) images (Fig. 4a) as well as lung image slices (Fig. 4b, c). In addition to the lungs, increased [^18^F]FDG uptake was observed in bone marrow, particularly in the sternum and vertebrae, which is a hallmark of marrow reactive hyperplasia commonly observed in individuals with active infections (Fig. 4b, c). Notably, [^18^F]FDG uptake in the marrow increased on days 4 and 6 post-infection. Analysis of VOIs uncovered a steady increase in lung [^18^F]FDG uptake from day 1 to day 6 post-infection. Compared to pre-infection, lung [^18^F]FDG uptake on day 1 increased 1.40-fold though not statistically significant (effect size, *d* = 2.51; critical *t*,* t*_*c*_ = 3.18) (Fig. 4d). Lung uptake continued to increase relative to mock infection as the disease progressed and was 1.99-fold higher on day 3 (*d* = 4.66; *p* = 0.007) and 2.58-fold higher on day 5 (*d* = 4.83; *p* = 0.003) relative to pre-infection (Fig. 4d). Metabolic lung volume (MLV)—the volume of lung with [^18^F]FDG uptake above a defined baseline threshold—also increased with disease severity. Notably, MLV provided a more sensitive measure for detecting lung inflammation following influenza infection. The [^18^F]FDG uptake threshold was established using lung uptake values from pre-infected and mock-infected mice. Consistent with overall lung uptake, MLV expanded in proportion to influenza disease severity (Fig. 4e). MLV in PR8-infected lungs increased 3.66-fold on day 1 (*d* = 3.31; *t*_*c*_ = 3.18; *p* = 0.008), 4.36-fold on day 3 (*d* = 5.82; *p* = 0.002), and 5.32-fold on day 5 (*d* = 4.09; *p* = 0.02) compared to pre-infection (Fig. 4f). In contrast, lung [^18^F]FDG uptake and MLV in mock-infected mice remained unchanged over time (Fig. 4d, f).

In addition to serial imaging, we also performed cross-sectional PET/CT imaging in another cohort of mice (Fig. 2). Compared to baseline, lung uptake was 1.59-fold higher on day 1 (*d* = 3.42; *t*_*c*_ = 2.23; *p* = 0.005) and increased to 2.67-fold on day 6 (*d* = 10. 9; *p* < 0.001) (Fig. 4 g). MLV expanded by 4.84-fold on day 1 post-infection (*d* = 2.98; *t*_*c*_ = 2.23; *p* = 0.02); and as high as 9.31-fold on day 6 (*d* = 7.14; *p* < 0.001) compared to baseline (Fig. 4h), in line with lung uptake. These findings corroborated the longitudinal PET imaging and *ex vivo* gamma counting data, which altogether confirmed that lung [^18^F]FDG uptake serves as a reliable *in vivo* imaging biomarker of influenza lung infection and inflammation. Furthermore, [^18^F]FDG uptake differentiates healthy from infected lungs as early as day 1 post-infection, whereas body weight loss only became apparent from day 3 (Fig. 1b).

**Fig. 5:**
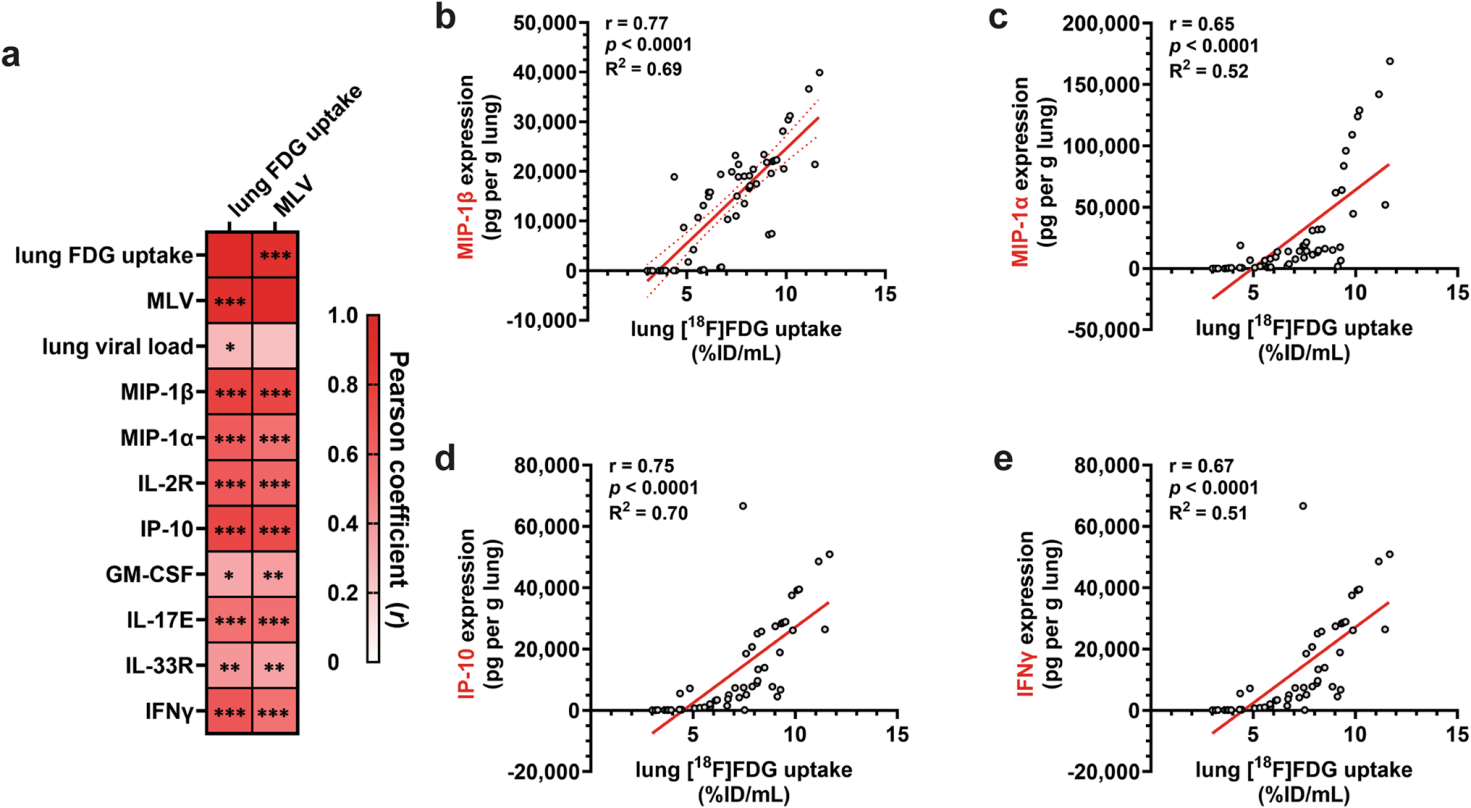
Correlation between lung [^18^F]FDG uptake and lung inflammation. **a** Heatmap of Pearson coefficient of the linear relationship between lung [^18^F]FDG uptake (%ID/mL) and various parameters. *p*-values are shown in individual cells. * *p* < 0.05. ** *p* < 0.005. *** *p* < 0.001. Individual scatter plots for lung uptake *vs.* expression of cytokines and chemokines: **b** MIP-1β, **c** MIP-1α, **d** IP-10, and **e** IFN-γ

To determine whether lung [^18^F]FDG uptake was a marker of viral replication or virus-mediated lung inflammation, we analyzed its correlation with various parameters using the cross-sectional PET/CT data from Cohort 3. Virus replication in the lungs (*i.e.,* viral load) showed only a weak correlation with lung [^18^F]FDG uptake and no correlation with MLV (Fig. 5a). In contrast, lung [^18^F]FDG uptake strongly correlated with the expression of inflammatory chemokines, including MIP-1β (Fig. 5a-b), MIP-1α (Fig. 5a, c), and IP-10 (CXCL10) (Fig. 5a, d), which are involved in granulocyte recruitment and neutrophilic inflammation. Additionally, [^18^F]FDG uptake was strongly correlated with IFN-γ expression (Fig. 5a, e). These data demonstrate that lung [^18^F]FDG uptake is a sensitive biomarker of the host’s inflammatory response to influenza infection and permits early detection of influenza-mediated lung inflammation.

### Lung [^18^F]FDG-PET Is More Sensitive Than Lung CT Imaging in Detecting Influenza Disease

**Fig. 6:**
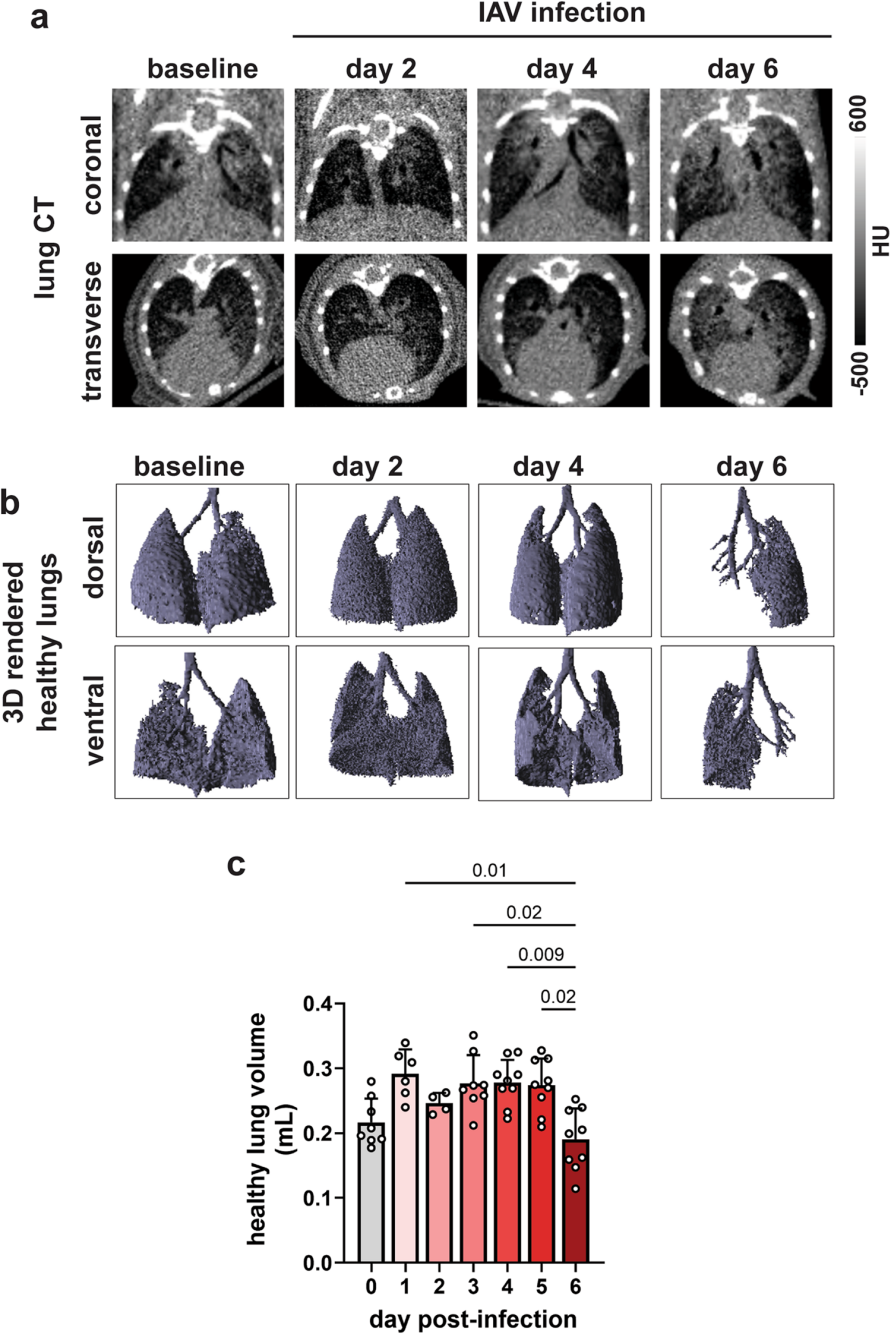
Detection of lung inflammation by CT imaging. **a** Coronal and transverse slices of influenza-infected lungs. **b** 3D surface-rendered images of healthy lung volumes measured on CT images. **c** Healthy lung volumes of influenza infected mice measured by VOI analysis on lung CT images. Data are shown as mean ± SEM (*n* = 9 mice/day) from three independent studies. Means were compared by Welch’s ANOVA

To assess the utility of lung CT imaging in detecting influenza-induced lung disease, we segmented healthy lung VOIs over several days of infection, as described in the Methods section. Visual inspection of transverse and coronal CT images did not clearly differentiate diseased from healthy lungs on days 2 and 4 post-infection (Fig. 4b, 6a). While X-ray attenuation in the lungs became more apparent on day 6 post-infection (Fig. 4b, 6a), CT imaging was unable to capture early disease progression. Further, 3D rendering of healthy lung volumes showed a significant reduction in healthy lung volume only on day 6 (the peak of disease) (Fig. 6b). Cross-sectional lung CT imaging confirmed a rapid decline of healthy lung volumes only on day 6 post-infection, relative to earlier time points (Fig. 6c). In contrast, lung [^18^F]FDG-PET scans detected inflammation as early as day 1 post-infection (Fig. 4d, f-h), well before any significant changes were observed on CT scans. The results demonstrate that [^18^F]FDG-PET is superior to CT imaging in detecting influenza-driven lung disease in murine models.

## Discussion

In this study, we evaluated lung [^18^F]FDG uptake as a robust non-invasive imaging biomarker of lung inflammation and disease in a murine model of influenza infection. Inoculation of influenza A (IAV) PR8 virus strain, a widely used mouse-adapted influenza strain, into a fully immunocompetent BALB/c mouse led to lethal disease marked by significant viral replication and inflammation in the lungs. Histopathological analysis revealed immune cell infiltration into the lung parenchyma and alveoli, along with upregulated expression of translocator protein (TSPO), an inflammatory marker highly expressed on myeloid-derived hematopoietic cells such as macrophages, monocytes, and neutrophils [34, 36]. The expression of pro-inflammatory cytokines, including MIP-1α (CCL3), MIP-1β (CCL-4), IP-10 (CXCL10), and IFN-γ, steadily increased and reflect the ongoing host immune response—particularly the recruitment and activation of granulocytes and monocytes to the lungs [37, 38]. These findings are consistent with previous studies on influenza PR8 mouse infection [39, 40] and highlight the escalating immune response and inflammation as disease progresses [41, 42].

Given the increased inflammatory activity in the lungs resulting from influenza infection, we hypothesized that lung uptake of [^18^F]FDG, a radioactive analogue of glucose, would increase due to heightened metabolic demand in the inflamed tissue. *Ex vivo* gamma counting confirmed this hypothesis and revealed a significant increase in [^18^F]FDG retention in the lungs as early as day 2 post-infection. Other organs, such as the heart, brain, liver, spleen, intestines, also exhibited elevated [^18^F]FDG which indicated systemic inflammation. Notably, we observed increased radioactivity in the blood on days 4 and 6 post infection, likely due to immune cell mobilization from the bone marrow to the infected lungs, as also seen in our dengue and Zika virus mouse infection models [23, 24].

To further assess the specificity of this [^18^F]FDG signal, we normalized tissue activity to residual blood activity in diseased lungs relative to healthy lungs (lung localization ratios, LR). Only the lungs manifested a clear, disease-dependent increase in [^18^F]FDG uptake, which suggests selective pulmonary metabolic enhancement during infection. While lymphoid tissues like the spleen and lymph nodes have been reported to accumulate [^18^F]FDG in response to viral infection [19, 21, 24, 34], we found minimal extra-pulmonary lymphoid involvement in our model. Increased [^18^F]FDG retention but not LR in these tissues indicated enhanced mobilization and transport of metabolically active inflammatory cells through the blood characteristic of systemic inflammation. Notably, we did not investigate [^18^F]FDG retention in intrapulmonary lymph nodes (mediastinal, subcarinal, *etc.*), which could also contribute to the observed lung signal. Nonetheless, prior studies reported pronounced neutrophil (Ly6G^+^) and cytotoxic-T cell (CD8^+^) infiltration, as well as a decrease in B cell (CD19^+^) counts in the spleen during influenza [43]. However, the phagocyte activation and spleen damage was minimal in the tissue [44]. Similarly, neutrophil counts were significantly elevated in bone marrow of influenza-infected mice at peak disease [45]. These cellular changes confirm active immunologic response in these tissues, despite limited [^18^F]FDG retention observed *ex vivo*.

We noted similarly increased uptake in lung [^18^F]FDG-PET/CT data from influenza-infected mice. Our results also demonstrate that lung [^18^F]FDG uptake serves as an early biomarker of influenza-mediated inflammation, detectable in PET scans as early as day 1 post-infection in both cross-sectional and serial imaging studies. Strong correlations between lung [^18^F]FDG uptake and the expression of pro-inflammatory cytokines that drive leukocyte recruitment to the lungs also confirm that [^18^F]FDG is a robust biomarker of host inflammation rather than direct viral replication. In contrast, clinical signs like body weight loss (beginning on day 3), and lung opacity on CT imaging (detectable only at day 6), lagged behind [^18^F]FDG uptake. These highlight the superior sensitivity of [^18^F]FDG-PET for early detection of lung disease. While [^18^F]FDG is a non-specific metabolic tracer, its ability to detect inflammatory lesions in this influenza model underscores its utility for monitoring host responses to influenza infection. While we did not evaluate inactivated virus, our pilot studies using self-limiting influenza B and rhinovirus-1B models—both of which do not exhibit overt signs of disease—revealed transient lung [^18^F]FDG uptake at early infection correlating with mild increase in inflammatory cytokines and chemokines in the lungs (data not shown). This suggests that [^18^F]FDG may still capture early inflammatory responses in the absence of severe pathology.

This study presents the first comprehensive investigation of [^18^F]FDG lung uptake in the well-established influenza A PR8 mouse model, offering new insights into the use of molecular imaging for viral lung infections. Our findings extend prior work by demonstrating that [^18^F]FDG-PET effectively captures lung inflammation and serves as an accurate and reliable measure of complex inflammatory processes *in vivo*. Collectively, our results validate lung [^18^F]FDG-PET uptake as a molecular imaging biomarker of inflammation in severe influenza infections.

A limitation of our study is the use of a highly lethal dose of influenza A PR8 virus, which induced severe disease and inflammatory lesions in the lungs, making detection by [^18^F]FDG-PET relatively straightforward. A lethal model was selected to represent patients with severe disease, for whom [^18^F]FDG-PET imaging may have greatest diagnostic value in the early identification of severe inflammation. However, lung [^18^F]FDG uptake in models of self-limiting influenza may also provide insights into lung inflammation and resulting morbidity.

A second caveat is that increased [^18^F]FDG activity in the blood, as seen in late disease *ex vivo*, also contributes to the uptake values in PET images. Distribution volume analysis from dynamic PET data would aid in distinguishing *in situ* lung [^18^F]FDG uptake from the contribution of blood from either increased blood perfusion or more rapid mobilization of metabolically active inflammatory cells in the blood [46, 47]. It should also be noted that no partial volume correction was applied in our study. Given the proximity of the lungs to both myocardium and activated bone marrow in the spine, signal spillover cannot be ruled out. However, the strong correlation between PET values and *ex vivo* [^18^F]FDG counts (Fig. S1) supports the accuracy of uptake trends.

Lastly, we are unable to differentiate in the PET images whether the observed [^18^F]FDG uptake was primarily due to infiltrating bone marrow-derived immune cells or due to increased metabolic activity in lung resident cells. Future studies should address these questions by evaluating the sensitivity of [^18^F]FDG-PET in models of varying disease severity and exploring cellular origins of the observed signal.

Looking ahead, [^18^F]FDG-PET imaging could be further explored for its potential to monitor the efficacy of antiviral or anti-inflammatory therapies in influenza infections. In this study, we have demonstrated that [^18^F]FDG-PET is a valuable tool for early detection and monitoring of lung inflammation, opening the door for future research into its clinical application for influenza and potential other viral disease of global significance.

## Supplementary Information

Below is the link to the electronic supplementary material.

**Fig. S1**. *Lung [*^*18*^*F]FDG uptake from serial PET imaging*. (**a**) Quantification of areas under the curve (AUC) values from lung [18F]FDG uptake trends reported in *Fig. 3h*. (**b**) *Ex vivo* gamma counts of [^18^F]FDG retained in lung tissues at necropsy and tissue localization ratio (LR). Data are shown as mean ± SD for mock (*n* = 5) and PR8-infected (*n* = 4) mice. Means were compared by Welch’s *t*-test. * *p* < 0.05. ** *p* < 0.005. (**c**) Linear relationship between lung [^18^F]FDG uptake in PET/CT images *vs*. lung [^18^F]FDG retention in gamma counting. *r*, Pearson coefficient. *R*^*2*^ = linear coefficient. (PNG 177 KB)

Supplementary file1 (TIF 1.36 MB)

## Data Availability

All research data are available from the corresponding author upon request.

## References

[1] Paget J, Staadegaard L, Wang X, Li Y, van Pomeren T, van Summeren J et al (2023) Global and national influenza-associated hospitalisation rates: estimates for 40 countries and administrative regions. J Glob Health 13:04003. 10.7189/jogh.13.04003 36701368 10.7189/jogh.13.04003 PMC9879557

[2] Lafond KE, Porter RM, Whaley MJ, Suizan Z, Ran Z, Aleem MA et al (2021) Global burden of influenza-associated lower respiratory tract infections and hospitalizations among adults: a systematic review and meta-analysis. PLoS Med 18:e1003550. 10.1371/journal.pmed.1003550 33647033 10.1371/journal.pmed.1003550 PMC7959367

[3] Cozza V, Campbell H, Chang HH, Iuliano AD, Paget J, Patel NN et al (2021) Global seasonal influenza mortality estimates: a comparison of 3 different approaches. Am J Epidemiol 190:718–727. 10.1093/aje/kwaa196 32914184 10.1093/aje/kwaa196 PMC8218989

[4] Group IPC (2024) Global burden associated with 85 pathogens in 2019: a systematic analysis for the global burden of disease study 2019. Lancet Infect Dis 24:868–895. 10.1016/S1473-3099(24)00158-0 38640940 10.1016/S1473-3099(24)00158-0 PMC11269650

[5] Goeijenbier M, van Sloten TT, Slobbe L, Mathieu C, van Genderen P, Beyer WEP et al (2017) Benefits of flu vaccination for persons with diabetes mellitus: a review. Vaccine 35:5095–5101. 10.1016/j.vaccine.2017.07.095 28807608 10.1016/j.vaccine.2017.07.095

[6] Hulme KD, Gallo LA, Short KR (2017) Influenza virus and glycemic variability in diabetes: a killer combination? Front Microbiol 8:861. 10.3389/fmicb.2017.00861 28588558 10.3389/fmicb.2017.00861 PMC5438975

[7] Ekstrand JJ (2012) Neurologic complications of influenza. Semin Pediatr Neurol 19:96–100. 10.1016/j.spen.2012.02.004 22889537 10.1016/j.spen.2012.02.004

[8] Chaves SS, Nealon J, Burkart KG, Modin D, Biering-Sorensen T, Ortiz JR et al (2023) Global, regional and national estimates of influenza-attributable ischemic heart disease mortality. EClinicalMedicine 55:101740. 10.1016/j.eclinm.2022.101740 36425868 10.1016/j.eclinm.2022.101740 PMC9678904

[9] Carbonell R, Moreno G, Martin-Loeches I, Bodi M, Rodriguez A (2023) The role of biomarkers in influenza and COVID-19 community-acquired pneumonia in adults. Antibiotics (Basel) 12(1):161. 10.3390/antibiotics12010161 10.3390/antibiotics12010161 PMC9854478 36671362

[10] Liu S, Huang Z, Deng X, Zou X, Li H, Mu S et al (2021) Identification of key candidate biomarkers for severe influenza infection by integrated bioinformatical analysis and initial clinical validation. J Cell Mol Med 25:1725–1738. 10.1111/jcmm.16275 33448094 10.1111/jcmm.16275 PMC7875920

[11] Danaher PJ, Phillips M, Schmitt P, Richard SA, Millar EV, White BK et al (2022) Breath biomarkers of influenza infection. Open Forum Infect Dis 9:ofac489. 10.1093/ofid/ofac489 36267247 10.1093/ofid/ofac489 PMC9578165

[12] Borras E, McCartney MM, Thompson CH, Meagher RJ, Kenyon NJ, Schivo M et al (2021) Exhaled breath biomarkers of influenza infection and influenza vaccination. J Breath Res. 10.1088/1752-7163/ac1a61 34343985 10.1088/1752-7163/ac1a61 PMC8763390

[13] Lakadamyali M, Rust MJ, Babcock HP, Zhuang X (2003) Visualizing infection of individual influenza viruses. Proc Natl Acad Sci USA 100:9280–9285. 10.1073/pnas.0832269100 12883000 10.1073/pnas.0832269100 PMC170909

[14] Heaton NS, Leyva-Grado VH, Tan GS, Eggink D, Hai R, Palese P (2013) In vivo bioluminescent imaging of influenza a virus infection and characterization of novel cross-protective monoclonal antibodies. J Virol 87:8272–8281. 10.1128/JVI.00969-13 23698304 10.1128/JVI.00969-13 PMC3719835

[15] Kim JH, Bryant H, Fiedler E, Cao T, Rayner JO (2022) Real-time tracking of bioluminescent influenza A virus infection in mice. Sci Rep 12:3152. 10.1038/s41598-022-06667-w 35210462 10.1038/s41598-022-06667-w PMC8873407

[16] Tran V, Moser LA, Poole DS, Mehle A (2013) Highly sensitive real-time in vivo imaging of an influenza reporter virus reveals dynamics of replication and spread. J Virol 87:13321–13329. 10.1128/JVI.02381-13 24089552 10.1128/JVI.02381-13 PMC3838222

[17] Czako R, Vogel L, Lamirande EW, Bock KW, Moore IN, Ellebedy AH et al (2017) In vivo imaging of influenza virus infection in immunized mice. MBio. 10.1128/mBio.00714-17 28559489 10.1128/mBio.00714-17 PMC5449660

[18] Jain SK (2017) The promise of molecular imaging in the study and treatment of infectious diseases. Mol Imaging Biol 19:341–347. 10.1007/s11307-017-1055-0 28155078 10.1007/s11307-017-1055-0 PMC5407939

[19] Dyall J, Johnson RF, Chen DY, Huzella L, Ragland DR, Mollura DJ et al (2011) Evaluation of monkeypox disease progression by molecular imaging. J Infect Dis 204:1902–1911. 10.1093/infdis/jir663 22013221 10.1093/infdis/jir663 PMC3209815

[20] Hayasaka D, Nishi K, Fuchigami T, Shiogama K, Onouchi T, Shimada S et al (2016) [^18^F]FDG PET imaging for identifying the dynamics of intestinal disease caused by SFTSV infection in a mouse model. Oncotarget 7:140–147. 10.18632/oncotarget.6645 26700962 10.18632/oncotarget.6645 PMC4807988

[21] Chefer S, Thomasson D, Seidel J, Reba RC, Bohannon JK, Lackemeyer MG et al (2015) Modeling [(^18^)F]-FDG lymphoid tissue kinetics to characterize nonhuman primate immune response to Middle East respiratory syndrome-coronavirus aerosol challenge. EJNMMI Res 5:65. 10.1186/s13550-015-0143-x 26573211 10.1186/s13550-015-0143-x PMC4646887

[22] Hartman AL, Nambulli S, McMillen CM, White AG, Tilston-Lunel NL, Albe JR et al (2020) SARS-CoV-2 infection of African green monkeys results in mild respiratory disease discernible by PET/CT imaging and shedding of infectious virus from both respiratory and gastrointestinal tracts. PLoS Pathog 16:e1008903. 10.1371/journal.ppat.1008903 32946524 10.1371/journal.ppat.1008903 PMC7535860

[23] Chacko AM, Watanabe S, Herr KJ, Kalimuddin S, Tham JY, Ong J et al (2017) 18F-fdg as an inflammation biomarker for imaging dengue virus infection and treatment response. JCI Insight. 10.1172/jci.insight.93474 28469088 10.1172/jci.insight.93474 PMC5414563

[24] Victorio CBL, Ong J, Tham JY, Reolo MJ, Novera W, Msallam R et al (2022) Preclinical evaluation of [(18)F]FDG-PET as a biomarker of lymphoid tissue disease and inflammation in Zika virus infection. Eur J Nucl Med Mol Imaging 49:4516–4528. 10.1007/s00259-022-05892-9 35876869 10.1007/s00259-022-05892-9 PMC9309455

[25] Focosi D, Caracciolo F, Galimberti S, Papineschi F, Petrini M (2008) False positive PET scanning caused by inactivated influenza virus vaccination during complete remission from anaplastic T-cell lymphoma. Ann Hematol 87:343–344. 10.1007/s00277-007-0413-4 18092164 10.1007/s00277-007-0413-4

[26] Iyengar S, Chin B, Margolick JB, Sabundayo BP, Schwartz DH (2003) Anatomical loci of HIV-associated immune activation and association with viraemia. Lancet 362:945–950. 10.1016/S0140-6736(03)14363-2 14511927 10.1016/S0140-6736(03)14363-2

[27] Shirone N, Shinkai T, Yamane T, Uto F, Yoshimura H, Tamai H et al (2012) Axillary lymph node accumulation on FDG-PET/CT after influenza vaccination. Ann Nucl Med 26:248–252. 10.1007/s12149-011-0568-x 22271546 10.1007/s12149-011-0568-x

[28] Intriago B, Danus M, Calvo N, Escobar J, Montero M, Kohan S et al (2009) Influenza-like infection can result in diffuse fluordeoxyglucose uptake in the lungs. Clin Nucl Med 34:737–738. 10.1097/RLU.0b013e3181b539f7 19893419 10.1097/RLU.0b013e3181b539f7

[29] Jonsson CB, Camp JV, Wu A, Zheng H, Kraenzle JL, Biller AE et al (2012) Molecular imaging reveals a progressive pulmonary inflammation in lower airways in ferrets infected with 2009 H1N1 pandemic influenza virus. PLoS ONE 7:e40094. 10.1371/journal.pone.0040094 22911695 10.1371/journal.pone.0040094 PMC3401186

[30] Camp JV, Bagci U, Chu YK, Squier B, Fraig M, Uriarte SM et al (2015) Lower respiratory tract infection of the ferret by 2009 H1N1 pandemic influenza A virus triggers biphasic, systemic, and local recruitment of neutrophils. J Virol 89:8733–8748. 10.1128/JVI.00817-15 26063430 10.1128/JVI.00817-15 PMC4524093

[31] Rodriguez L, Nogales A, Martinez-Sobrido L (2017) Influenza A Virus Studies in a Mouse Model of Infection. J Vis Exp. 10.3791/55898 28930978 10.3791/55898 PMC5752200

[32] Victorio CB, Xu Y, Ng Q, Chua BH, Alonso S, Chow VT et al (2016) A clinically authentic mouse model of enterovirus 71 (EV-A71)-induced neurogenic pulmonary oedema. Sci Rep 6:28876. 10.1038/srep28876 27357918 10.1038/srep28876 PMC4928123

[33] van Elden LJ, Nijhuis M, Schipper P, Schuurman R, van Loon AM (2001) Simultaneous detection of influenza viruses A and B using real-time quantitative PCR. J Clin Microbiol 39:196–200. 10.1128/JCM.39.1.196-200.2001 11136770 10.1128/JCM.39.1.196-200.2001 PMC87701

[34] Victorio CBL, Msallam R, Novera W, Ong J, Yang TJ, Ganasarajah A et al (2023) Tspo expression in a Zika virus murine infection model as an imaging target for acute infection-induced neuroinflammation. Eur J Nucl Med Mol Imaging 50:742–755. 10.1007/s00259-022-06019-w 36348095 10.1007/s00259-022-06019-w PMC9852192

[35] Morais JA, Longo J, Pacheco T (2017) Lung volume segmentation for preclinical studies using Albira CT and PMOD software. Bruker BioSpin

[36] Victorio CBL, Ganasarajah A, Novera W, Ong J, Msallam R, Chacko AM (2024) Translocator protein (TSPO) is a biomarker of Zika virus (ZIKV) infection-associated neuroinflammation. Emerg Microbes Infect 13:2348528. 10.1080/22221751.2024.2348528 38662785 10.1080/22221751.2024.2348528 PMC11132733

[37] Menten P, Wuyts A, Van Damme J (2002) Macrophage inflammatory protein-1. Cytokine Growth Factor Rev 13:455–481. 10.1016/s1359-6101(02)00045-x 12401480 10.1016/s1359-6101(02)00045-x

[38] Lu X, Masic A, Liu Q, Zhou Y (2011) Regulation of influenza A virus induced CXCL-10 gene expression requires PI3K/Akt pathway and IRF3 transcription factor. Mol Immunol 48:1417–1423. 10.1016/j.molimm.2011.03.017 21497908 10.1016/j.molimm.2011.03.017

[39] Fukushi M, Ito T, Oka T, Kitazawa T, Miyoshi-Akiyama T, Kirikae T et al (2011) Serial histopathological examination of the lungs of mice infected with influenza A virus PR8 strain. PLoS ONE 6:e21207. 10.1371/journal.pone.0021207 21701593 10.1371/journal.pone.0021207 PMC3118813

[40] Zarnegar B, Mendez-Enriquez E, Westin A, Soderberg C, Dahlin JS, Gronvik KO et al (2017) Influenza infection in mice induces accumulation of lung mast cells through the recruitment and maturation of mast cell progenitors. Front Immunol 8:310. 10.3389/fimmu.2017.00310 28382037 10.3389/fimmu.2017.00310 PMC5360735

[41] Riviere F, Burger J, Lefevre F, Garnier A, Vigne C, Tournier JN et al (2023) Infection with influenzavirus A in a murine model induces epithelial bronchial lesions and distinct waves of innate immune-cell recruitment. Front Immunol 14:1241323. 10.3389/fimmu.2023.1241323 37649477 10.3389/fimmu.2023.1241323 PMC10464834

[42] Hufford MM, Richardson G, Zhou H, Manicassamy B, Garcia-Sastre A, Enelow RI et al (2012) Influenza-Infected Neutrophils within the Infected Lungs Act as Antigen Presenting Cells for Anti-Viral CD8+ T Cells. PLoS ONE 7:e46581. 10.1371/journal.pone.0046581 23056353 10.1371/journal.pone.0046581 PMC3466305

[43] Ancicova L, Wagnerova M, Janulikova J, Chalupkova A, Hrabovska Z, Kostolansky F et al (2015) Simultaneous infection with gammaherpes and influenza viruses enhances the host immune defense. Acta Virol 59:369–379. 10.4149/av_2015_04_369 26666185 10.4149/av_2015_04_369

[44] Sun S, Zhao G, Xiao W, Hu J, Guo Y, Yu H et al (2011) Age-related sensitivity and pathological differences in infections by 2009 pandemic influenza A (H1N1) virus. Virol J 8:52. 10.1186/1743-422X-8-52 21299904 10.1186/1743-422X-8-52 PMC3041774

[45] Donovan C, Thorpe AE, Yarak R, Coward-Smith M, Pillar AL, Gomez HM et al (2024) Maternal thirdhand exposure to e-cigarette vapor alters lung and bone marrow immune cell responses in offspring in the absence or presence of influenza infection. Am J Physiol Lung Cell Mol Physiol 327:L796–L806. 10.1152/ajplung.00078.2024 39316673 10.1152/ajplung.00078.2024

[46] Muzi M, O’Sullivan F, Mankoff DA, Doot RK, Pierce LA, Kurland BF et al (2012) Quantitative assessment of dynamic PET imaging data in cancer imaging. Magn Reson Imaging 30:1203–1215. 10.1016/j.mri.2012.05.008 22819579 10.1016/j.mri.2012.05.008 PMC3466344

[47] Tamaki N, Hirata K, Kotani T, Nakai Y, Matsushima S, Yamada K (2023) Four-dimensional quantitative analysis using FDG-PET in clinical oncology. Jpn J Radiol 41:831–842. 10.1007/s11604-023-01411-4 36947283 10.1007/s11604-023-01411-4 PMC10366296

