# Supplementary figures and images for: Validation of Lung [18F]FDG Uptake as a Quantitative PET Biomarker for Influenza-Associated Pulmonary Inflammation
Source: Mol Imaging Biol. 2025 Nov 5;27(6):930–42. doi: 10.1007/s11307-025-02051-y (PMC12804336; doi:10.1007/s11307-025-02051-y)

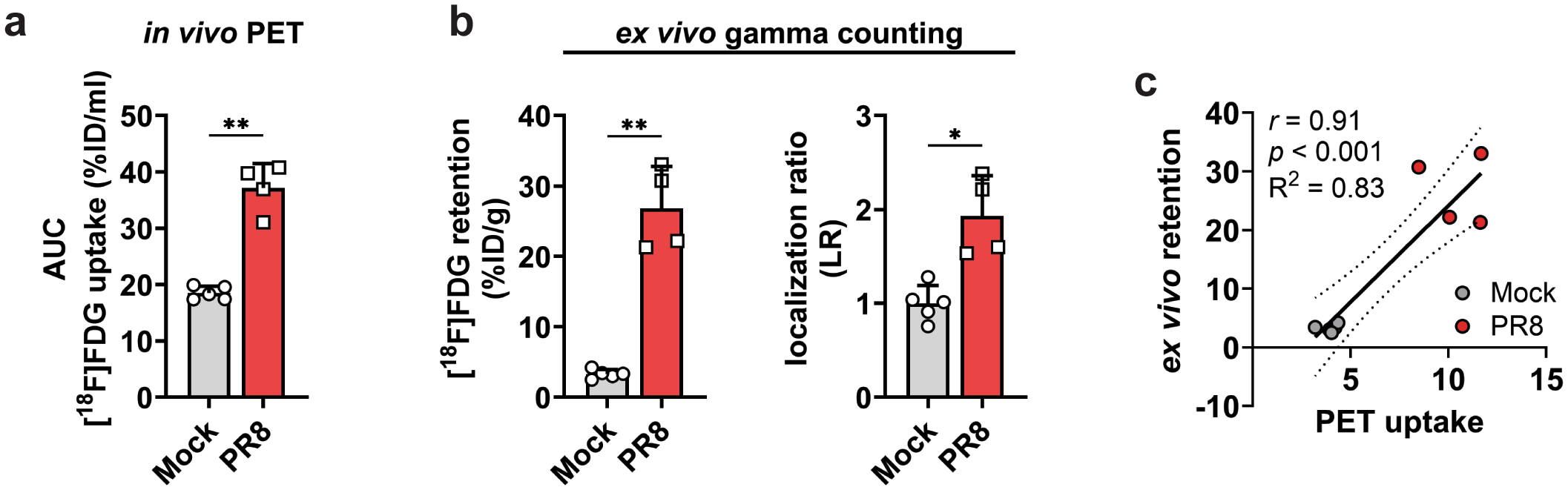

Supplement: Supplementary file 1 — Fig. S1. Lung [18F]FDG uptake from serial PET imaging. (a) Quantification of areas under the curve (AUC) values from lung [18F]FDG uptake trends reported in Fig. 3h. (b) Ex vivo gamma counts of [18F]FDG retained in lung tissues at necropsy and tissue localization ratio (LR). Data are shown as mean ± SD for mock (n = 5) and PR8-infected (n = 4) mice. Means were compared by Welch’s t-test. * p < 0.05. ** p < 0.005. (c) Linear relationship between lung [18F]FDG uptake in PET/CT images vs. lung [18F]FDG retention in gamma counting. r, Pearson coefficient. R2 = linear coefficient. (PNG 177 KB) [file 11307_2025_2051_Fig7_ESM.png]

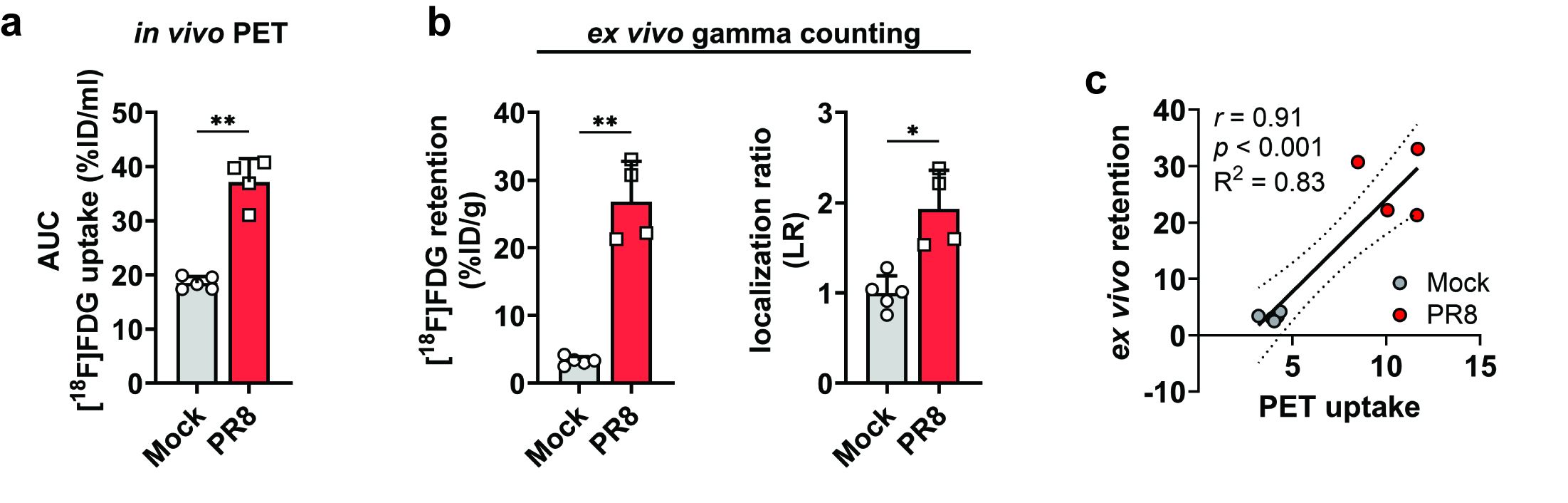

Supplement: Supplementary file 2 — Supplementary file1 (TIF 1.36 MB) [file 11307_2025_2051_MOESM1_ESM.tif]
